# New data on *Thelohanellus nikolskii* Achmerov, 1955 (Myxosporea, Myxobolidae) a parasite of the common carp (*Cyprinus carpio*, L.): The actinospore stage, intrapiscine tissue preference and molecular sequence

**DOI:** 10.1016/j.ijppaw.2021.04.004

**Published:** 2021-04-16

**Authors:** Réka Borzák, Muhammad Hafiz Borkhanuddin, Gábor Cech, Kálmán Molnár, Sascha L. Hallett, Csaba Székely

**Affiliations:** a Institute for Veterinary Medical Research, Centre for Agricultural Research, POB 18, H-1581, Budapest, Hungary; b Faculty of Science & Marine Environment, Universiti Malaysia Terengganu, 21030, Kuala Nerus, Malaysia; c Department of Microbiology, Oregon State University, Nash Hall 226, Corvallis, OR 9 7331, USA

**Keywords:** Myxozoa, *Thelohanellus nikolskii*, Common carp, Myxospore, Actinospore, SSU rDNA, Cnidaria

## Abstract

*Thelohanellus nikolskii*, Achmerov, 1955 is a well-known myxozoan parasite of the common carp (*Cyprinus carpio* L.). Infection regularly manifests in numerous macroscopic cysts on the fins of two to three month-old pond-cultured carp fingerlings in July and August. However, a *Thelohanellus* infection is also common on the scales of two to three year-old common carp in ponds and natural waters in May and June. Based on myxospore morphology and tissue specificity, infection at both sites seems to be caused by the same species, namely *T. nikolskii*. This presumption was tested with molecular biological methods: SSU rDNA sequences of myxospores from fins of fingerlings and scales of older common carp were analysed and compared with each other and with related species available in GenBank. Sequence data revealed that the spores from the fins and scales represent the same species, *T. nikolskii*. Our study revealed a dichotomy in both infection site and time in *T. nikolskii*-infections: the fins of young carp are infected in Summer and Autumn, whereas the scales of older carp are infected in Spring. Myxosporean development of the species is well studied, little is known, however about the actinosporean stage of *T. nikolskii.* A previous experimental study suggests that aurantiactinomyxon actinospores of this species develop in *Tubifex tubifex,* Müller, 1774. The description included spore morphology but no genetic sequence data (Székely et al., 1998). We examined >9000 oligochaetes from Lake Balaton and Kis-Balaton Water Reservoire searching for the intraoligochaete developmental stage of myxozoans. Five oligochaete species were examined, *Isochaetides michaelseni* Lastochin, 1936, *Branchiura sowerbyi* Beddard, 1892, *Nais* sp., Müller, 1774, *Dero* sp. Müller, 1774 and *Aelosoma* sp. Ehrenberg, 1828. Morphometrics and SSU rDNA sequences were obtained for the released actinospores. Among them, from a single *Nais* sp., the sequence of an aurantiactinomyxon isolate corresponded to the myxospore sequences of *T. nikolskii*.

## Highlights

• *Thelohanellus nikolskii* is well known from fins of common carp; molecular analyses confirmed infections also on the scales.
• Histological analyses demonstrated that *Thelohanellus nikolskii* is a chondrophil myxosporean.
• *Thelohanellus nikolskii* exhibits a dichotomy in infection site and time in common carp.
• *Thelohanellus nikolskii* infects fins of young carp in Summer and Autumn, and the scales of older carp in Spring.
• The SSU rDNA of an aurantiactinomyxon released from a *Nais* sp. corresponded to *Thelohanellus nikolskii.*

## Graphical abstract

**Figure:**
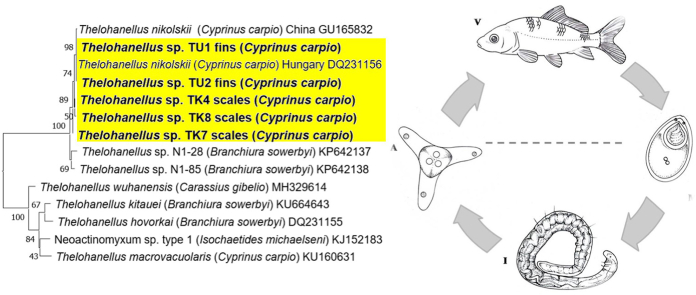

## 1 Introduction

*Thelohanellus nikolskii* Achmerov, 1955 is a common, pathogenic myxosporean parasite specific to common carp. Originating in the Far East, it was inadvertently introduced to Europe in 1979 where it subsequently caused heavy infection in fingerlings of common carp cultured in fish ponds (Jeney, 1979; Molnár and Kovács-Gayer, 1981–1982; Molnár, 1982; Čirkovic et al., 2013). Achmerov (1955, 1960) was the first to describe four *Thelohanellus* species (*Thelohanellus nikolskii*, *Thelohanellus amurensis*, *Thelohanellus dogieli* and *Thelohanellus hovorkai*) from Amur wild carp (*Cyprinus carpio haematopterus*) in the Amur Basin. Of these, *T. nikolskii* infected the fins, while *T. amurensis* Achmerov, 1955, *T. dogieli* Achmerov, 1955 and *T. hovorkai* Achmerov, (1960) parasitised the liver, skin and abdominal cavity, respectively. Two other *Thelohanellus* species showed high morphological similarity to *T. nikolskii*: *Thelohanellus cyprini* Hoshina and Hosoda (1957) infects the fins and *Thelohanellus kitauei* Egusa and Nakajima (1981) infects the intestines and skin of common carp (Zhai et al., 2016). Recently, *T. cyprini* was proved to be a synonym of *T. nikolskii* (Zhang et al., 2013).

In Hungary, *Thelohanellus* infection in common carp fry was first detected by Jeney (1979) who identified the species as *T. dogieli*. However, accepting Achmerov’s (1955) theory on the strict tissue specificity of *Thelohanellus* spp., Molnár and Kovács-Gayer (1981, 1982) and Molnár (1982) later identified the parasite as *T. nikolskii*. Histological study of plasmodia developing in the cartilaginous tissue of the fin rays was done by Molnár (1982), while the ultrastructure on the sporogenesis of this species was studied by Desser et al. (1983). In the synopsis of Zhang et al. (2013), 11 other *Thelohanellus* species, some of them with questioned validity, have been reported from different organs of the common carp: *T. acuminatus* Achmerov, 1955; *T. callisporis*, Ky, 1971; *T. chuhsinensis* Ma, Dong and Ma, 1999; *T. hokiagensis* Ma, Dong and Wang, 1999; *T*. *kyi* (Ky 1971) Zhang et al., (2013); *T. leshanensis* Zhao and Ma, 1992; *T. parasagittarius* Chen and Ma, 1998; *T. pekingensis* Chen and Ma, 1998; *T. quinghoensis* Li and Wen, 1992; *T. sagittarius* Lie and Nie, 1973 and *T. wananensis* Lei, 1988.

Jeney (1979) supposed that *Thelohanellus* infection of the common carp was introduced to Hungary through importation of *Cyprinus carpio haematopterus* (Amur wild carp, koi carp), a subspecies of the common carp, from the Far East. Besides Hungary, *T. nikolskii* infection in the fins of carp fingerlings became a wide ranging disease in several European countries, e.g., in Serbia (Čirkovic, 1986; Hacmanjek, 1985), in Czechia (Dyková and Lom, 1988), and in Moldova (Trombitsky et al., 1983, 1990; Moshu, 1993). Thelohanellosis on the scales of older carp specimens were observed first by Moshu and Molnár (1997) in Moldova and soon after the infection was observed also in Hungary (Molnár and Székely, 1997). Based on morphological similarity of myxospores and cysts and development of plasmodia in the cartilaginous tissue of the scales and fins, the above authors identified the species developing in the scales also as *T. nikolskii*.

Known life cycles of myxosporeans include development in an invertebrate host. The intraoligochaete development of *T. nikolskii* was studied first by Székely et al. (1998). These authors experimentally infected the oligochaetes *Branchiura sowerbyi* Beddard, 1892 *Tubifex tubifex* Müller, 1774 and *Limnodrilus hoffmeisteri* Claparède, 1862 with *T. nikolskii* myxospores, and detected waterborne aurantiactinomyxon type actinospores released from *Tubifex tubifex*, however this connection has not been confirmed with molecular genetic data.

Our study confirms the identity of *Thelohanellus nikolskii-*like cysts developing in the fins and scales of common carp, using a combination of morphological, histological and molecular genetic methods. Moreover, the oligochaete host and the actinospore type of *T. nikolskii* was determined by studying actinosporeans released by oligochaetes from Lake Balaton and Kis-Balaton Reservoire.

Common carp (*Cyprinus carpio*) in Hungary is cultured in ponds according to a three year system. After artificial propagation in a hatchery, carp fry are raised in a series of nursery ponds, whereas two or three year-old stocks are reared in large, often over 100 ha, ponds. Natural propagation of carp in natural waters is negligible, due to the absence of suitable spawning grounds. Natural waters are resupplied with two and three year old carp year after year from fish farms.

## 2 Materials and methods

### 2.1 Collection of fish and myxospore samples

Two month-old common carp fingerlings with fin infections with *T. nikolskii* cysts were collected in July 2015 from a fish pond where about 70% of the stock was infected by this parasite. Earlier in the same year, seven specimens of three-year-old common carp with scale infections of *Thelohanellus* plasmodia were also collected. Three specimens were collected from the Kis-Balaton Water Reservoir in May and June while four specimens were obtained from fish farms throughout the country in the same period (Table 1). Infected fish were transported to the laboratory alive in plastic sacs filled with oxygen. Fingerlings in the laboratory were kept for some days in aquaria in aerated water whereas older carp were placed in concrete basins supplied with flowing water. Before dissection, a drop of clove oil was added to the water for sedating the fish, then they were killed by a cervical cut, in accordance with ACUP protocols.

**Table 1:** Summary of the data about *Thelohanellus* spp. infected fish samples examined with molecular methods in this study.

Name of the sample	Age of the carp	Organ	Sampling date (dd.mm.yyyy)	Sampling site	Location in Hungary
TU1	Fingerling	fins	14.07.2015	Tiszagyála	North-East Hungary
TU2	Fingerling	fins	14.07.2015	Tiszagyála	North-East Hungary
TK4	3 years old	scales	19.05.2015	Kis-Balaton Reservoire	West Hungary
TK5	3 years old	scales	01.06.2015	Tiszavasvári	North-East Hungary
TK6	3 years old	scales	02.06.2015	Kőrösladány	South-East Hungary
TK7	3 years old	scales	03.06.2015	Kis-Balaton Reservoire	West Hungary
TK8	3 years old	scales	04.06.2015	Ráckeve	Central Hungary
TK9	3 years old	scales	10.06.2015	Kis-Balaton Reservoire	West Hungary
TK10	3 years old	scales	12.06.2015	Paks	South Hungary

Plasmodia were collected from the fins of carp fry and from the scales of older specimens under a dissectrion microscope. Myxospores obtained from plasmodia were characterised according to the guidelines of Lom and Arthur (1989). Spores both from fin cysts or scales were checked under light microscope, measured and compared with previous data available in this laboratory (Molnár 1982; Molnár and Székely 1997). Measurements of myxospores in frontal view were taken from microphotograph of a thin layer of spores under a coverslip with a calibrated microscope. Spores were also measured in sutural view in less thin preparations. Thirty myxospores collected from two plasmodia were measured from fin cyst, and 15 myxospores were measured from scale cysts, each from the samples collected in the fish Farm Tiszagyála, and in the Kis-Balaton water reservoir.

For histological comparison of fin and scale infections, one sample from each tissue was collected. Tail fin was cut from a two month old, pond cultured common carp fingerling, which was heavily infected (about 50 cysts) with *Thelohanellus nikolskii* plasmodia. Infected scales were removed from a single fish selected from the older fish samples. Scales containing two or three plasmodia were fixed in Bouin's solution, embedded in paraffin wax, cut to 4–5 μm sections, and after dehydration in ethanol series and aceton, stained with haematoxylin and eosin. Histological sections were photographed with an Olympus DP10 digital camera.

For molecular studies, myxospores were collected from two fingerlings infected with more than 50 plasmodia. From both fish, a single fin plasmodium containing more than 5000 spores was selected and after opening the plasmodium with a needle, spores were preserved in 80% alcohol (TU1, TU2) until further studies. From each of the three year-old fish a single plasmodium (TK4, TK5, TK6, TK7, TK8, TK9, TK10) was selected from the scales for the comparative molecular analysis (Table 1).

### 2.2 Collection of oligochaetes

Sediment from Lake Balaton and Kis-Balaton Reservoire with previous *T. nikolskii* records was collected near water vegetation at about 0.5–1 m depth with a net in 2010–2015. On each sampling occasion, as much as 40–60 L of mud was sieved *in-situ* through a 1000 μm mesh net that removed clay particles. Oligochaetes trapped together with debris, vegetation roots and decayed particles were then transferred to the laboratory with minimal lake water. Additional dechlorinated tap water and aeration were supplied in the laboratory for the collection. Oligochaetes were hand-sorted from the retained material in trays filled with dechlorinated tap water. Additionally, the retained material was placed onto a large-sized mesh sieve (500–1000 μm) immersed in dechlorinated tap water up to the level of mesh of the sieve for several hours or overnight. This encouraged the oligochaetes to make their way into the water in the trays through the mesh of the sieves. Oligochaetes were identified according to the key to Timm (1999). Some questionable specimens were identified by Tarmo Timm (Vörstjarv Limnological Institute, Estonia) through photo or couriered specimens. Oligochaetes were collected throughout the year except during Winter (November to March).

### 2.3 Morphological investigation of actinospore types released

Oligochaetes were separated into cell-well-plates according to the methods of Yokoyama et al. (1991). They were placed individually in each cell well with 200 μl dechlorinated tap water. Plates were then kept at room temperature (23–25 °C) and stored at 4 °C. Each well was scanned for released actinospores using a Zeiss Treval 3 inverted microscope. Released actinospores were pipetted, examined with a compound microscope and several preserved in 80% ethanol for molecular identification. Photomicrographs were taken from fresh actinospores under both bright and phase contrast field, using a DP-20 digital camera mounted on an Olympus BH-2 microscope. Subsequently, line drawings of actinospores were made based on the photos.

Measurements of the morphological characteristics were taken from a variable number of spores (depending on availability) from one infected oligochaete if possible. Measurements of actinospores were made according to the guidelines of Lom et al. (1997). The number of secondary cells and turns of tubules in polar capsules is given for certain actinospores where the number could be confidently determined by light microscopy. Prevalence of infection of the *T. nikolskii* actinospore type was calculated based on the percentage of infected *Nais* sp. A sample of released actinospores from each infected oligochaete was preserved in 80% ethanol.

### 2.4 Molecular identification of the collected spores

Myxo- and actinospore samples preserved in ethanol from fish and infected oligochaetes were centrifuged at 10,000×*g* for 10 min, then the ethanol removed. The genomic DNA was extracted from the pelleted spores using the DNeasy® Blood & Tissue Kit (Qiagen, Hilden, Germany) according to the manufacturer's instructions. The partial SSU rDNA region was amplified using a nested PCR described in detail by Cech et al. (2015); universal eukaryotic primers ERIB1 and ERIB10 (Barta et al., 1997) were used in the first round PCR and myxozoan-specific primers Myx1F and SphR (Hallett and Diamant, 2001; Eszterbauer and Székely, 2004) were used in the second round PCR.

The amplicons were analysed by electrophoresis in a 1% agarose gel. All the appropriate PCR products were excised from the gel, purified with the Gel/PCR DNA Fragments Extraction Kit (Geneaid, New Taipei City, Taiwan) and sequenced directly using the BigDye Terminator v3.1 Cycle Sequencing Kit (Life Technologies) with an ABI PRISM® 3100 Genetic Analyser (Life Technologies). The following primers were used for the sequencing reaction to generate overlapping fragments and coverage in both directions: ACT1fr, MC3, MC5, MB5r, MB3f, SphR and CR1 R (Hallett and Diamant, 2001; Molnár et al., 2002; Eszterbauer and Székely, 2004; Székely et al., 2015).

The sequence fragments were assembled using MEGA 7 software (Kumar et al., 2016). The contiguous SSU rDNA sequences and the most similar myxozoan sequences from GenBank based on BLAST matches were aligned with the software CLUSTAL W (Thompson et al., 1994). DNA pairwise distances were calculated with the MEGA 7 software using the Maximum Composite Likelihood model. Phylogenetic analysis was performed on a 1704 bp final alignment via Maximum Likelihood (ML) with *Myxobolus cerebralis* as the outgroup. The dataset was tested using MEGA 7 for the nucleotide substitution model of best fit and the model, shown by the Akaike Information Criterion (AIC) as the best-fitting one, was chosen (GTR + G + I model). Bootstrap values based on 1000 resampled datasets were generated.

## 3 Results

### 3.1 General observations of Thelohanellus nikolskii infections in Hungary

**Fig. 1:**
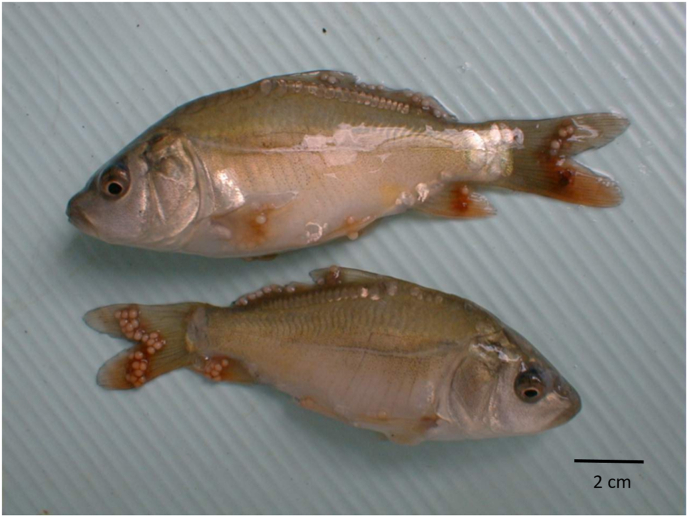
*Thelohanellus nikolskii* cysts on the fins of carp fingerlings.

**Fig. 2:**
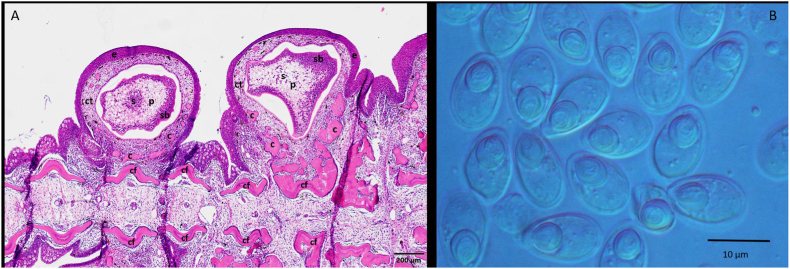
A: Section of an infected fin, containing *T. nikolskii* cysts, stained with hematoxilin-eosin. Cartilage of finray (cf) is next to the cyst. Plasmodium (p) is in the achromatic tegument, mature myxospores (s) are in the middle, sporoblasts (sb) are at the edges. Around the plasmodium, there is a thick connective tissue (ct) layer, containing cartilaginous elements (c). Multilayer epithelium (e) is the outer layer. B: *T. nikolskii* myxospores from the plasmodium.

**Fig. 3:**
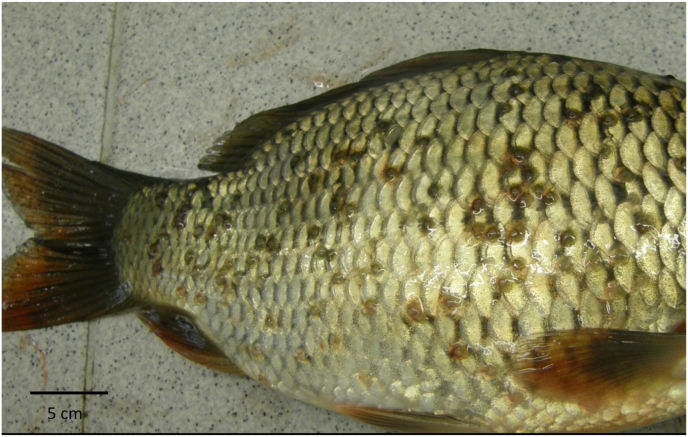
*Thelohanellus* cysts on the scales of an aged common carp specimen.

**Fig. 4:**
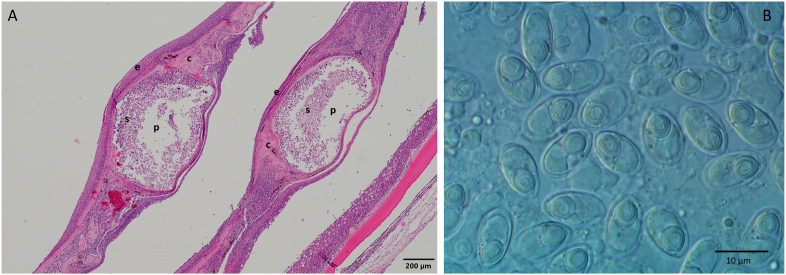
A: Cross section of infected scales, stained with hematoxilin-eosin. The plasmodia (p) are filled with myxospores (s) and are surrounded by cartilaginous tissue (c) of the scales, covered by the epithelium layer (e). B: *T. nikolskii* myxospores from a plasmodium in the scale.

*Thelohanellus nikolskii* infection was observed in carp fry cultured in ponds from the second half of July to September (Summer) (Fig. 1). The first external sign of infection was darkening of the fin and appearance of dark colour nodules in the fin-rays of 3–4 cm long carp. In some fish the fins were eroded (see Molnár and Kovács-Gayer, 1981–1982; Molnár, 1982). Mature plasmodia filled with myxospores appeared at the end of July and early August (Fig. 2). By the end of August most myxospores were released from opened plasmodia and in the autumn months only distortions of the fins marked past infections. Less frequently, late formation of cysts were recorded also in September in 8–16 cm long fingerlings. The ultrastructure of the spores corresponded to *T. nikolskii* spores described by Desser et al. (1983). In two year-old carp mostly scale infections were observed but less frequently fin infections also occurred. In the three year-old carp *Thelohanellus* plasmodia were found only in the scales. These plasmodia infected series of scales causing roughness on the surface of fish (Fig. 3). Concurrent infections on the scales and fins were not recorded. The earliest scale-thelohanellosis was observed at the beginning of May (spring) and the latest one was recorded in the middle of June. In these infections, plasmodia were located at the outer periphery of the scales, in the non-overlapping region. The plasmodium was surrounded by a very thin cartilaginous layer. The original compact cartilaginous plate of the scale was damaged and only calcified islands could be recognised (Fig. 4A). The ultrastructure of the plasmodia and the myxospores obtained from the scales (Fig. 4AB) corresponded to those described by Moshu and Molnár (1997). The myxospores obtained from fin and scale cysts had a similar shape and overlapped in measurements (Table 2).

**Table 2:** Comparison of infection site, size of plasmodia and myxospore dimensions of Thelohanellus nikolskii collected from the fins and scales of common carp. All measurements except the size of plasmodia are in micrometer (μm).

Localisation	Size of plasmodia (mm)	Spore length	Spore width	Spore thickness	Capsule length	Capsule width	Polar tubule turns	Thickness of valves	Reference
**Fin rays**	**Up to 2**	**17.2 ± 1.5 (15.6**–**19.0)**	**10.6 ± 1 (8.7**–**12.5)**	**8.6 ± 1.1 (7.5**–**10)**	**6.8 ± 0.6 (6.2**–**7.5)**	**5.3 ± 0.7 (4.7**–**5.2)**	**6**–**7**	**0.7**–**1**	**this study**
Fins	Up to 2	16.5 (15.0–19.0)	10.0 (8.7–12.5)	8.7 (7.5–10.0)	6.5 (6.2–7.5)	5.6 (4.7–6.2)	6–7	1.2–2.2	Moshu and Molnár (1997)
Fins	Up to 2	17.2 (13.4–20.6)	10.8 (9.2–13.3)	n.d.	6.5 (5.8–7.6)	5.9 (5.2–6.8)	n.d.	n.d.	Circovic et al. 2013
**Scales**	**Up to 2**	**16.2 ± 1.8 (14.0**–**18.5)**	**9 ± 1.6 (7.5**–**11.2)**	**6.6 ± 0.6 (6.2**–**7.5)**	**6.3 ± 0.4 (5.8**–**6.6)**	**5.4 ± 0.8 (4.8**–**6.5)**	**6**–**7**	**0.7**–**1**	**this study**
Scales	Up to 3	17.5 (16.2–18.7)	10.0 (7.5–11.2)	7.5 (6.2–7.5)	7.5 (6.2–7.5)	5.6 (5.0–6.5)	6–7	1.2–1.5	Moshu and Molnár (1997)
Scales	Up to 3	17.7 (13.5–20.3)	10.9 (10–12.1)	n.d.	7.1 (6.1–8.7)	6.2 (5.8–6.6)	n.d.	n.d.	Circovic et al. 2013

### 3.2 Actinospore stage of Thelohanellus nikolskii in oligochaetes

**Fig. 5:**
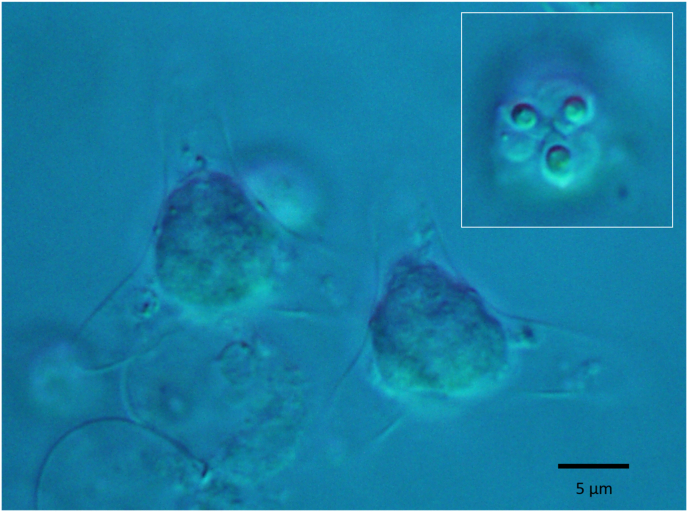
Microphotograph of fresh, unstained actinospore of Aurantiactinomyxon type (AUM5) from *Nais* sp. Insert – apical view of spore with protruding polar capsules.

**Fig. 6:**
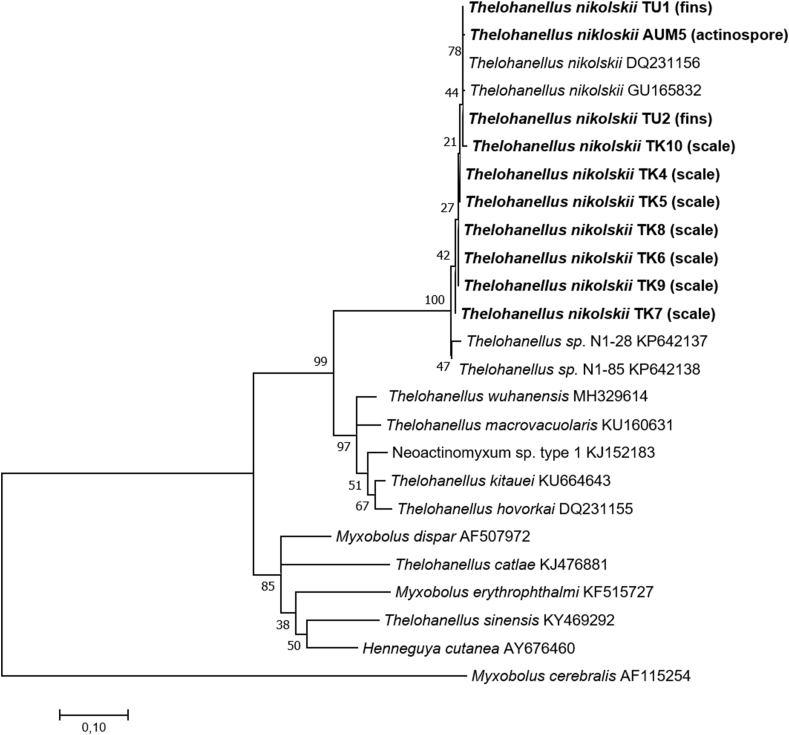
Phylogenetic position of *Thelohanellus nikolskii* spores from the fins and scales of common carp based on SSU rDNA analysis by the Maximum Likelihood algorithm. *Myxobolus cerebralis* was used as the outgroup. Bootstrap values are given at the nodes. The scale-bar indicates the number of expected substitutions per site.

Altogether 9452 oligochaetes were collected, belonging to the five most common oligochaete species in the Lake: *Isochaetides michaelseni* Lastochin, 1936, *Branchiura sowerbyi* Beddard, 1892, *Nais* sp., Müller, 1774, *Dero* sp. Müller, 1774 and *Aelosoma* sp. Ehrenberg, 1828. Thirteen different actinospore morphotypes were identified from *I. michaelseni*, *B. sowerbyi* and *Nais* sp. However, no actinospores were found from *Dero* and *Aelosoma* spp. Actinospores could be assigned to the aurantiactinomyxon (5), neoactinomyxum (1), raabeia (2), synactinomyxon (1), and triactinomyxon (4) collective groups. Aurantiactinomyxon types were the most diverse form while triactinomyxon types were the most prevalent (Table 3). One *Nais* sp. specimen out of the 1200 examined released an undescribed aurantiactinomyxon type actinospore (Fig. 5), which corresponded to *T. nikolskii* based on the SSU rDNA sequence (Fig. 6). Only these spores were the subject of this study and are described as follows:

**Table 3:** Summary of actinospore morphotypes detected by the authors in the sampling period from 2010 to 2015.

Morphotype	Host	Prevalence	Reference
Aurantiactinomyxon 1	*Isochaetides michaelseni*	10/7818	Borkhanuddin, 2013 (dissertation)
Aurantiactinomyxon 2	*Isochaetides michaelseni*	6/7818	Borkhanuddin, 2013 (dissertation)
Aurantiactinomyxon 3	*Branchiura sowerbyi*	1/434	Borkhanuddin, 2013 (dissertation)
Aurantiactinomyxon 4	*Branchiura sowerbyi*	1/434	Zhao et al. (2016)
**Aurantiactinomyxon 5**	***Nais* sp.**	**1/1200**	**this study**
Neoactinomyxum 1	*Isochaetides michaelseni*	2/7818	Borkhanuddin et al. 2014
Raabeia 1	*Isochaetides michaelseni*	5/7818	Borkhanuddin et al. 2014
Raabeia 2	*Isochaetides michaelseni*	2/7818	Borkhanuddin et al. 2014
Synactinomyxon 1	*Isochaetides michaelseni*	1/7818	Borkhanuddin et al. 2014
Triactinomyxon 1	*Isochaetides michaelseni*	NA	Székely et al., 2014
Triactinomyxon 2	*Isochaetides michaelseni*	NA	Székely et al., 2014
Triactinomyxon 3	*Isochaetides michaelseni*	NA	Székely et al., 2014
Triactinomyxon 4	*Isochaetides michaelseni*	NA	Borkhanuddin, 2013 (dissertation)

Aurantiactinomyxon type

*Description*: Mature actinospores (n = 10) triangular to almost circular in apical view, diameter 10.3 (9.3–12.0) μm. Caudal processes of equal length, finger-like with rounded tips, and curved downwards, 14.6 (12.7–16.0) μm and 6.5 (5.3–7.3) μm wide, with largest span of 36.9 (33.5–40.2) μm. Polar capsules, three equally sized, round in apical view, 3.3 × 2.6 μm, with 3–4 rounds of polar tubule. Sporoplasm contains 8 germ cells.

*Type host*: *Nais* sp*.*

*Type locality:* Kis Balaton.

*Prevalence of infection*: 1/1200 (0.08%)

*Site of infection:* Body coelom.

*Molecular data:* An SSU rDNA amplicon of 477 bp was generated (GenBank accession # MT569892). A BLAST search indicated highest similarity (99.8%) with *T. nikolskii* (DQ231156), differing in one nucleotide.

*Remarks*: Actinospores were found floating in the water of the cell-well plate in which the *Nais* worm was kept.

*Differential diagnosis*: This Aurantiactinomyxon type differs from other aurantiactinomyxons by having shorter caudal processes, about half to one-third of the length of known types. The caudal processes dimensions were most similar to the aurantiactinomyxon type ‘B2’ of Eszterbauer et al. (2006).

### 3.3 Molecular biological data

Partial SSU rDNA sequences were analysed from myxospores from the fins of fingerlings (two myxospore samples TU1, TU2), from myxospores from the scales of three year old common carp specimens (seven samples TK4, TK5, TK6, TK7, TK8, TK9, TK10) and from actinospore samples released by *I. michaelseni*, *B. sowerbyi* and *Nais* sp. Results of the released actinospores by the first two oligochaete species have been already published (Borkhanuddin et al. 2014; Zhao et al., 2016). The Aurantiactinomyxon morphotype released by the *Nais* sp. became a part of this study (AUM5). The sequence lengths and GenBank accession numbers with pairwise distances to *T. nikolskii* (DQ231156) are presented in Table 4. SSU rDNA sequences >1000 bp were generated from samples TU1, TU2, TK4, TK5, TK6, TK7, TK8 and TK9, and shorter sequences were determined from TK10 and the actinospore sample (AUM5) which were used to display the phylogenetic positions of the examined myxozoans on the Maximum Likelihood tree (Fig. 6). All of the aligned sequences were 99.0%–100% similar to the previously deposited SSU rDNA sequences of *T. nikolskii* (DQ231156 and GU165832) in GenBank. Based on the final 1704 bp alignment, the conserved region was 1070 bp, the variable region was 573 bp and the parsimony informative region was 344 bp. The mean distance between the fin (2 samples) and scale (7 samples) sample groups was 0.4%. Within the groups, the overall mean distances were 0.4% in scale samples and 0.1% in fin samples.

**Table 4:** Details of myxosporean samples sequenced (SSU rDNA) and similarity to *Thelohanellus nikolskii*.

Sample name	Spore type	Source	Sequence length (bp)	GenBank accession number	Similarity to *T. nikolskii* (DQ231156)
TU1	myxospores	fins of carp fingerlings	1618	MT535575	100%
TU2	myxospores	fins of carp fingerlings	1271	MT535576	100%
TK4	myxospores	scales of 3 year old carp	1618	MT535578	99.6%
TK5	myxospores	scales of 3 year old carp	1618	MT535580	99.6%
TK6	myxospores	scales of 3 year old carp	1618	MT535581	99.4%
TK7	myxospores	scales of 3 year old carp	1618	MT535577	99.0%
TK8	myxospores	scales of 3 year old carp	1618	MT535579	99.4%
TK9	myxospores	scales of 3 year old carp	1618	MT535582	99.4%
TK10	myxospores	scales of 3 year old carp	592	MT535583	99.3%
AUM 5	actinospores	oligochaete - *Nais*	477	MT569892	99.8%

## 4 Discussion

Most myxosporeans, among them *Thelohanellus* spp. are host, organ and tissue specific (Molnár, 1994; Molnár and Eszterbauer, 2015). For example, Achmerov (1960) reported four different *Thelohanellus* species from the Amur wild carp each of them infecting a different organ. However, Borzák et al. (2018) pointed out that some myxosporeans can develop also in different organs if the organ is composed of the same tissue. The occurrence of *Thelohanellus* plasmodia with myxospores in scales of older common carp (2–3 year olds), corresponding in size and shape to *T. nikolskii* described originally from fin rays of carp fingerlings, suggested that this myxosporean species might develop in two different sites in common carp depending on host age. Notably, the tissue preference of plasmodia in the two different locations was the same, namely plasmodia started their development in the cartilaginous elements of the fins and scales built up from collagenous material (Moshu and Molnár, 1997). Consideration of the formation of the cartilage in fin rays and in scales provides insight into this apparent dichotomy of infection site. In general it is accepted that fin rays are made from cartilage. However, there are no chondrocytes in the fin rays contrary to the rays in gill lamellae. Collagenous material of fin rays is produced by perichondreal (periosteal) cells (Molnár, 1982; Desser et al., 1983). As the fish gets older the collagenous fin ray becomes rigid, losing its soft structure due to ossification and in this process becomes less suitable for plasmodial development. On the other hand, histology of scale infection with myxosporeans is relatively less studied. In scale infections with *T. nikolskii* serious scale deformation was observed. The cartilaginous scale plate fragmented into amorphous cartilage islands, and the plasmodia developed in the cartilaginous elements. The plasmodia on the scales were located in non-overlapping regions which are particularly exposed to external effects. After minor injuries the scales start regenerating quickly, however the calcification process depends on external conditions, like temperature (Ghods et al., 2020), calcium and phosphate content, and salinity of the water (Ogawa et al., 2010). Until calcification is complete, the regenerating scale also has a soft structure, which might facilitate the infection with *T. nikolskii*.

During the development of *T. nikolskii*, beside the different organ location, there is also an apparent temporal dichotomy. Myxospores in older carp individuals developed in the springtime while those in fingerlings were formed during the summer or autumn. A similar phenomenon was observed by Circovic et al. (2013) who studied the prevalence and different forms of *T. nikolskii* in Serbia. One of the two possible explanations for the different sporulation times is that fingerlings and older fishes are cultured in separate ponds in Hungary where the chance of cross-infections seems to be excluded. However, ponds receive water from the same channels which may carry actinospores. Moreover, different age groups of carp are often moved between ponds even in the same year which could provide relatively constant infection of oligochaetes and fish. Carp spawning in nature is usually from the end of April through May in Hungary. The larvae and the fry are very fragile so a parasite infection on the fin can easily cause host death, before any clinical signs appear. Usually in a month, they become fingerlings, when *T. nikolskii* cysts can be observed on the fins. Plasmodium development in older fish can happen earlier because they are already present in the ponds and the infection is not lethal.

Another possible explanation for the observed temporal dichotomy is that two distinct periods may exist during the development of *T. nikolskii*. There is one developmental phase from May to September and a second one from September to May. Myxospores released from fin cysts in September sink to the sediment and are consumed by oligochaetes in which intraoligochaete development culminates in the production of actinospores in about April (Spring). These actinospores could start developing plasmodia in two or three year-old carp during May. On the other hand, myxospores that developed on scales during May infect oligochaetes releasing actinospores subsequently around June and initiate infection of carp fingerlings. These two cycles seem to be asynchronous. This is consistent with the dependence of intraoligochaete development on water temperature and associated thermal temperature units or degree days (Székely et al., 1998; Marton and Eszterbauer 2012).

Molecular biological examinations based on SSU rDNA sequences were carried out in the present study which revealed that myxospores from fin cysts and scale cysts are almost identical. There is only 0.4% nucleotide differences between the scale and fin samples, comparable to the variance of the scale sequences, for which 0.4% dissimilarity also was observed within that group. Only 0.1% difference was observed among the fin samples. SSU rDNA sequences were used for identification which is a commonly used region in phylogenetic studies for Myxozoa. Their repetitive arrangement within the genome provides multiple templates of DNA for PCR. Despite the several DNA repair mechanisms, polymorphisms between repeats can occur even at significant levels (Weitemier et al., 2015), which can cause sequence differences among the individuals of the same species. The fin samples analysed in this study originated from the same fingerling population of a pond whereas the scale samples were taken from different carp populations throughout the country. Furthermore, the sample size of the sequenced myxospores from fins was low, compared to the scale samples. These conditions can explain the above mentioned sequence differences between the samples from different locations. The 0.4% difference between the two *Thelohanellus* groups collected from scales and fins fits into the general species concept for myxosporean species; i.e. > 2% interspecific SSU rDNA variation for the Myxosporea and, and 0.0%–3.6% intraspecific variation (summarised by Atkinson et al., 2015). However, in our study the mean distance within the group of fin samples was lower (0.1%) than the mean distance between groups (0.4%), and the overall genetic distance does not exceed the generally accepted distance values.

**Fig. 7:**
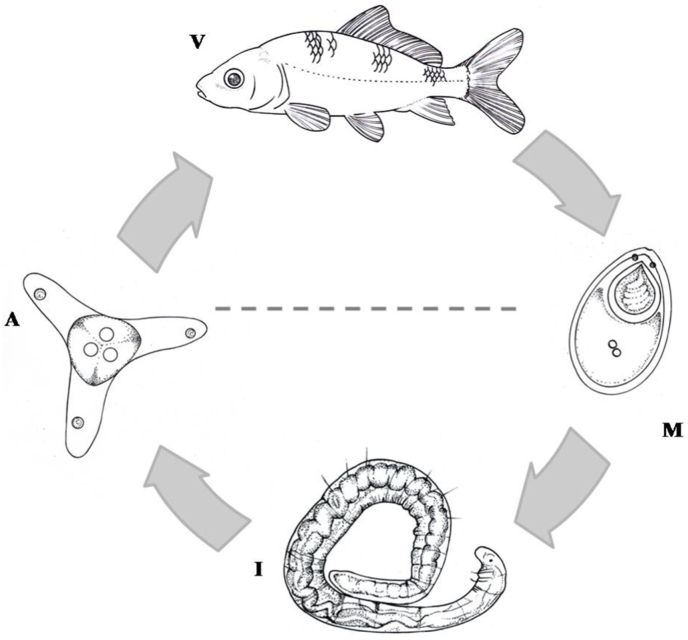
Schematic illustration of *T. nikolskii* life cycle: Aurantiactinomyxon-type actinospores (A) infect the vertebrate host *C. carpio* (V) in which they develop myxospores (M) that infect the invertebrate host *Nais* sp. (I).

During our oligochaete survey, we isolated several actinospore morphotypes, of which a short SSU rDNA sequence of an aurantiactinomyxon type released by a single unidentified *Nais* sp. was 99.8% similar to a myxospore sample collected from the fins of common carp, identified as *T. nikolskii* (DQ231156) by Eszterbauer et al., (2006). Thus our molecular comparison suggested that this aurantiactinomyxon represents the alternate life stage of *T. nikolskii* (Fig. 7). No genetic analysis was performed on the host *Nais* worm collected in this study; its identification was based on morphology. Out of the 1200 individuals of the *Nais* sp. that were monitored, only one worm released actinospores. Low infection prevalences in oligochaete populations are common in actinospores studies (e.g. Hallett et al., 2001; Rocha et al., 2020).

Aurantiactinomyxon morphotypes are known developmental stages for several *Thelohanellus* species, including *T. hovorkai* and *T. nikolskii* (Székely et al., 1998) and *Thelohanellus kitauei* (Zhao et al., 2016). The aurantiactinomyxon isolated in this study was morphologically distinct from that reported by Székely et al. (1998). Additionally, we collected the actinospores from a *Nais* sp. rather than a *Tubifex tubifex*. Since the earlier aurantiactinomyxon was characterised phenotypically only, a comparison of the DNA sequence data between the two studies was not possible. Notably, different phenotypes that correspond to a single genotype have been reported for aurantiactinomyxon (Hallett et al., 2002). However, a feature common to both phenotypes was the number of sporoplasm nuclei, which was not the case for the two aurantiactinomyxons associated with *T. nikolskii*. We suggest that the developing phase of *T. nikolskii* may occur in a range of oligochaete species, for which more broad research is needed to determine this association. Apart from aurantiactinomyxon, *Nais* worms have been reported to be the host for triactinomyxon-type actinospores for several myxosporean species such as *Triactinomyxon naidanum* (Naidu 1956), *Hoferellus cyprini* (Großheider and Körting, 1992), *Hoferellus carassii* (Trouillier et al., 1996), and *Myxobilatus gasterostei* (Atkinson and Bartholomew, 2009).

Oligochaete species belonging to Naidinae undergo reproduction in which several daughter worms are produced through a double cleavage process. Because naidines can reproduce rapidly every few days, they are able to reach high population densities quickly (Bely and Wray, 2004). This breeding feature enables myxozoan parasites to be transmitted via both horizontal and vertical pathways (Atkinson and Bartholomew, 2009). However, this propagation was not observed during our study, suggesting that an experimental study is needed to confirm this transmission pattern.

## Compliance with ethical standards

All experiments and handling of fish in the present study were conducted according to the animal welfare guidelines and recommendations (permission number PEI/001/1002–13/2015) under the Veterinary Medical Research Institute, Hungarian Academy of Sciences, Budapest, Hungary.

## References

[bib1] Achmerov A. (1955). Ways of the origin of Myxosporidia species of the genus *Thelohanellus* Kudo from Amur wild carp. Dokl. Akad. Nauk SSSR.

[bib2] Achmerov A. (1960). Myxosporidia of fish in the basin of river Amur.

[bib4] Atkinson S.D., Bartholomew J.L. (2009). Alternate spore stages of *Myxobilatus gasterostei*, a myxosporean parasite of three-spined sticklebacks (*Gasterosteus aculeatus*) and oligochaetes (*Nais communis*). Parasitol. Res..

[bib5] Atkinson S.D., Bartošová-Sojková P., Whipps C.M., Bartholomew J.L., Okamura B. (2015). Approaches for characterising myxozoan species. Myxozoan Evolution, Ecology and Development.

[bib6] Barta J.R., Martin D.S., Libetator P.A., Dashkevicz M., Anderson J.W., Feighner S.D., Elbrecht A., Perkins-Barrow A., Jenkins M.C., Danforth H.D., Ruff M.D., Profous-Juchelka H. (1997). Phylogenetic relationships among eight *Eimeria* species infecting domestic fowl inferred using complete small subunit ribosomal DNA sequences. J. Parasitol..

[bib7] Bely A.E., Wray G.A. (2004). Molecular phylogeny of naidid worms (Annelida: Clitellata) based on cytochrome oxidase I. Mol. Phylogenet. Evol..

[bib8] Borzák R., Molnár K., Cech G., Székely C. (2018). *Myxobolus* infection in the cornea of the roach (*Rutilus rutilus*) in Lake Balaton. Acta Vet. Hung..

[bib9] Cech G., Borzák R., Molnár K., Székely C. (2015). Three new species of *Myxobolus* Bütschli, 1882 (Myxozoa: Myxobolidae) infecting the common nase *Chondrostoma nasus* (L.) in the river Danube. Syst. Parasitol..

[bib10] Čirkovic M. (1986). Myxosporidiosis of the Common Carp Fingerlings.

[bib11] Čirkovic M., Novakov N., Aleksic N., Jovanovic M., Ljubojevic D., Babic R., Radosavljevic V. (2013). Different manifestations of *Thelohanellus nikolskii* infection in carp (*Cyprinus carpio*). Acta Vet..

[bib12] Desser S.S., Molnár K., Weller I. (1983). Ultrastructure of sporogenesis of *Thelohanellus nikolskii*, Achmerov, 1955 (Myxozoa, Myxosporea) from the common carp, *Cyprinus carpio*. J. Parasitol..

[bib13] Dyková I., Lom J. (1988). Review of pathogenic myxosporeans in intensive culture of carp (*Cyprinus carpio*) in Europe. Folia Parasitol..

[bib15] Egusa S., Nakajima K. (1981). A new Myxozoa *Thelohanellus kitauei*, the cause of intestinal giant cystic disease of carp. Fish Pathol..

[bib16] Eszterbauer E., Marton S., Rácz O.Z., Letenyei M., Molnár K. (2006). Morphological and genetic differences among actinosporean stages of fish-parasitic myxosporeans (Myxozoa): difficulties of species identification. Syst. Parasitol..

[bib17] Eszterbauer E., Székely C. (2004). Molecular phylogeny of the kidney parasitic *Sphaerospora renicola* from common carp (*Cyprinus carpio*) and *Sphaerospora* sp. from goldfish (*Carassius auratus auratus*). Acta Vet. Hung..

[bib20] Ghods S., Waddell S., Weller E., Renteria C., Jiang H.Y., Janak J.M., Mao S.S., Linley T.J., Arola D. (2020). On the regeneration of fish scales: structure and mechanical behavior. J. Exp. Biol..

[bib21] Großheider G., Körting W. (1992). First evidence that *Hoferellus cyprini* (Doflein, 1898) is transmitted by *Nais* sp. Bull. Eur. Assoc. Fish Pathol..

[bib22] Hacmanjek M. (1985). Thelohanellosis, a very frequent disease of Yugoslavian fish farms. Ribarstvo Jugoslavije.

[bib23] Hallett S.L., Atkinson S.D., El-Matbouli M. (2002). Molecular characterisation of two aurantiactinomyxon (Myxozoa) phenotypes reveals one genotype. J. Fish. Dis..

[bib24] Hallett S.L., Diamant A. (2001). Ultrastructure and small-subunit ribosomal DNA sequence of *Henneguya lesteri* n. sp. (Myxosporea), a parasite of sand whiting *Sillago analis* (Sillaginidae) from the coast of Queensland, Australia. Dis. Aquat. Org..

[bib25] Hallett S.L., Erséus C., O'Donoghue P.J., Lester R.J.G. (2001). Parasite fauna of Australian marine oligochaetes. Memoir. Queensl. Mus..

[bib26] Hoshina T., Hosoda S. (1957). On a new myxosporidian species, *Thelohanellus cyprini* n. sp., parasitic in the fin of *Cyprinus carpio*. J. Tokyo Univ. Fish.

[bib27] Jeney G. (1979). The occurrence of *Thelohanellus dogieli* Achmerov, 1955 (Myxosporidia) on carp (*Cyprinus carpio*) in fish ponds in Hungary. Parasitol. Hung..

[bib28] Kumar S., Stecher G., Tamura K. (2016). MEGA7: molecular Evolutionary genetics analysis version 7.0 for bigger datasets. Mol. Biol. Evol..

[bib29] Lom J., McGeorge J., Feist S.W., Morris D., Adams A. (1997). Guidelines for the uniform characterisation of the actinosporean stages of the phylum Myxozoa. Dis. Aquat. Org..

[bib31] Molnár K. (1982). Biology and Histopathology of *Thelohanellus nikolskii* Achmerov, 1955 (Myxosporea, Myxozoa), a Protozoan parasite of the common carp (*Cyprinus carpio*). Parasitol. Res..

[bib32] Molnár K. (1994). Comments on the host, organ and tissue specificity of fish myxosporeans and on the types of their intrapiscine development. Parasitol. Hung..

[bib33] Molnár K., Eszterbauer E., Okamura B., Gruhl A., Bartolomew J.L. (2015). Specificity of infection sites in vertebrate hosts. Myxozoan Evolution, Ecology and Development.

[bib34] Molnár K., Eszterbauer E., Székely C., Dán Á., Harrach B. (2002). Morphological and molecular biological studies on intramuscular *Myxobolus* spp. of cyprinid fish. J. Fish. Dis..

[bib35] Molnár K., Kovács-Gayer É. (1981–1982). Occurrence of two species of *Thelohanellus* (Myxosporea: Myxozoa) of Far-East origin in common carp populations of the Hungarian fish farms. Parasitol. Hung..

[bib36] Molnár K., Székely Cs (1997). *Thelohanellus nikolskii* infection on the scales of common carp in natural waters. (*Thelohanellus nikolskii* fertőzöttség természetes-vízi pontyok pikkelyzetén). Halászat..

[bib37] Moshu A. (1993). Myxosporidia of cultured fishes in Moldovian fish farms.

[bib38] Moshu A., Molnár K. (1997). *Thelohanellus* (Myxozoa: Myxosporea) infection of the scales in the European wild carp *Cyprinus carpio* carpio. Dis. Aquat. Org..

[bib39] Naidu K.V. (1956). A new species of actinomyxid sporozoan parasitic in a fresh-water oligochaete. J. Eukaryot. Microbiol..

[bib40] Ogawa N., Ura K., Takagi Y. (2010). Scale calcification in the goldfish in vitro: histological and quantitative analysis. Fish. Sci..

[bib42] Rocha S., Rangel L.F., Casal G., Azevedo C., Rodrigues P., Santos M.J. (2020). Involvement of sphaeractinomyxon in the life cycle of mugiliform-infecting *Myxobolus* (Cnidaria, Myxosporea) reveals high functionality of actinospore morphotype in promoting transmission. Parasitology.

[bib44] Székely C., El-Mansy A., Molnár K., Baska F. (1998). Development of *Thelohanellus hovorkai* and *Thelohanellus nikolskii* (Myxosporea: Myxozoa) in oligochaete alternate hosts. Fish Pathol..

[bib45] Székely C., Cech G., Chaudhary A., Borzák R., Singh H.S., Molnár K. (2015). Myxozoan infections of the three Indian major carps in fish ponds around Meerut, UP, India, with descriptions of three new species, *Myxobolus basuhaldari* sp. n., *M. kalavatiae* sp. n. and *M. meerutensis* sp. n., and the redescription of *M. catlae* and *M. bhadrensis*. Parasitol. Res..

[bib46] Thompson J.D., Higgins D.G., Gibson T.J. (1994). Clustal W: improving the sensitivity of progressive multiple sequence alignment through sequence weighting, position-specific gap penalties and weight matrix choice. Nucleic Acids Res..

[bib47] Timm T. (1999). Naturalist Handbooks I: A Guide to the Estonian Annelida.

[bib48] Trombitsky I.D., Golovina N.A., Sheinin M.E. (1983). Thelohanellosis of common carp. E I CNIIITEIRH.

[bib49] Trombitsky I.D., Sheinin M.E., Manja V.M. (1990). Biology and pathogenicity of *Thelohdnellus nikolskii*. Parazitologiya.

[bib50] Trouillier A., El-Matbouli M., Hoffmann R.W. (1996). A new look at the life-cycle of *Hoferellus carassii* in the goldfish (*Carassius auratus auratus*) and its relation to “kidney enlargement disease” KED. Folia Parasitol..

[bib51] Weitemier K., Straub S.C., Fishbein M., Liston A. (2015). Intragenomic polymorphism among high copy loci: a genus-wide study of nuclear ribosomal DNA in Asclepias (Aposynaceae). PeerJ.

[bib52] Yokoyama H., Ogawa K., Wakabayashi H. (1991). A new collection method of actinosporeans—a probable infective stage of myxosporeans to fishes—from tubificids and experimental infection of goldfish with the actinosporean, Raabeia sp. Fish Pathol..

[bib54] Zhai Y., Gu Z., Guo Q., Liu Y. (2016). New type of pathogenicity of *Thelohanellus kitauei* Egusa & Nakajima, 1981 infecting the skin of common carp *Cyprinus carpio L*. Parasitol. Int..

[bib55] Zhao D., Borkhanuddin M.H., Wang W., Liu Y., Cech G., Zhai Y., Székely C. (2016). The life cycle of *Thelohanellus kitauei* (Myxozoa: Myxosporea) infecting common carp *(Cyprinus carpio)* involves aurantiactinomyxon in *Branchiura sowerbyi*. Parasitol. Res..

[bib56] Zhang J.Y., Gu Z.M., Kalavati C., Eiras J.C., Liu Y., Guo Q.Y., Molnár K. (2013). A synopsis of the species of *Thelohanellus* Kudo, 1933 (Myxozoa: Myxosporea: Bivalvulida). Syst. Parasitol..

